# Guard Ring Design to Prevent Edge Breakdown in Double-Diffused Planar InGaAs/InP Avalanche Photodiodes

**DOI:** 10.3390/ma16041667

**Published:** 2023-02-16

**Authors:** Yu-Chun Chen, Ruei-Hong Yan, Hsu-Chia Huang, Liang-Hsuan Nieh, Hao-Hsiung Lin

**Affiliations:** 1 Graduated Institute of Electronics Engineering, National Taiwan University, Taipei 10617, Taiwan; 2 Graduated Institute of Photonics and Optoelectronics, National Taiwan University, Taipei 10617, Taiwan; 3 Department of Electrical Engineering, National Taiwan University, Taipei 10617, Taiwan

**Keywords:** APD, guard ring, InGaAs, InP, SAGCM, double-diffusion, edge breakdown, photonic device

## Abstract

We report on the design of an attached guard ring (AGR) and a floating guard ring (FGR) in a planar separate absorption, grading, charge, and multiplication In_0.53_Ga_0.47_As/InP avalanche photodiode to prevent premature edge breakdowns. The depths of the two Zn diffusions were utilized to manipulate the guard ring structures. Results from TCAD simulation indicate that the optimal AGR diffusion depth is right at the turning point where the breakdown current shifts from the edge of active region to the AGR region. The devices with optimal AGR depth contain significantly higher breakdown voltages than those of devices either with shallower—or without any— AGR. For the FGR design, a series of devices with different spacings between AGR and FGR and different FGR opening widths for diffusion were fabricated and characterized. We show that when the spacing is longer than the critical value, the breakdown voltage can increase ~1.5 V higher than those of the APD devices without FGR. In addition, the wider the FGR opening width, the higher the breakdown voltage. TCAD simulations were also performed to study the effect of FGR, but showed less pronounced improvements, which could be due the discrepancy between the calculated and experimental diffusion profile.

## 1. Introduction

In_0.53_Ga_0.47_As/InP avalanche photodetectors (APDs) measuring 1550 nm and exhibiting separate absorption, grading, charge, and multiplication (SAGCM) have been widely used in fiber communication and eye-safe LIDAR. The devices are employ the elegant SAGCM structure [1,2] in which multiplication and absorption can be separately designed and consist of materials which are lattice-matched to InP substrates, avoiding the effects of strain or defects. The devices can be operated in linear mode to amplify the detected light signal by impact ionization in the InP multiplication region or in the Geiger mode [3,4] to obtain a considerable signal resulting from the breakdown triggered by a few received photons. For InGaAs/InP SAGCM APDs, Zn diffusion is commonly used to form a planar InP p^+^-n junction. The diffusion depth further determines the thickness of the InP multiplication layer and thus, the breakdown behavior. However, the lateral diffusion may form a cylindrical edge junction, resulting in higher electric fields than those in the central active region. This high electric field may increase the dark current and cause premature breakdown in the edge region, which limits the electric field and multiplication gain in the active region. To mitigate this edge breakdown, two guard ring structures have been proposed in the literature [5,6,7,8,9,10,11,12]. The first structure uses additional Zn diffusions, with different diffusion depths, to surround the active region to directly reshape the lateral curvature of the cylindrical edge junction of the active region. This is commonly known as a junction extension [5], extended guard ring [6], or attached guard ring (AGR). The second example uses Zn diffusion to form isolated ring-shaped diffusion junctions surrounding the active region and is known as floating guard ring (FGR) [7,8,9,10]. This structure utilizes the depletion region of FGR to affect that of the edge junction of the active region to relieve the electric field and prevent edge breakdown.

This work reports the AGR and FGR design for a 1550 nm SAGCM In_0.53_Ga_0.47_As/InP planar APD device with dual Zn-diffusion. The AGR and FGR structures were formed through dual diffusions. The effect of three parameters relevant to the formation of AGR and FGR—the diffusion depth of AGR, the spacing between AGR and FGR, and the opening width of FGR—on the breakdown behavior was studied through both TCAD simulations and experimental work. Their optimum and critical values are addressed, and the mechanisms behind the behaviors are presented.

## 2. Experimental Methods

**Figure 1:**
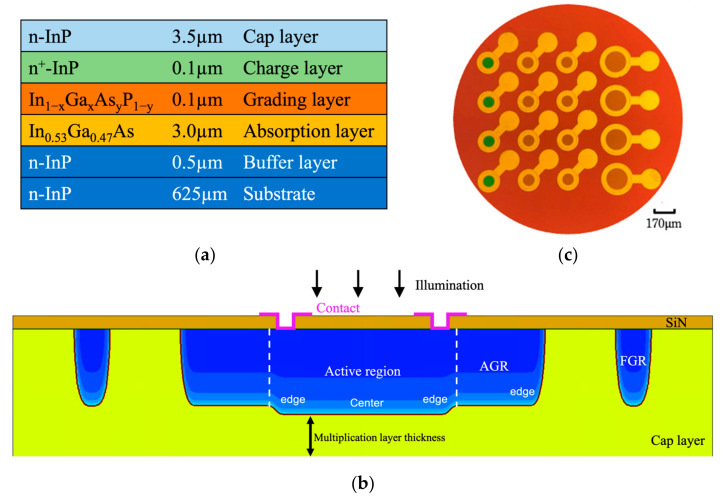
(**a**) Epitaxial layer structure of SAGCM APD; (**b**) schematic diagram showing the active region, AGR, and FGR, formed by Zn diffusions in the cap layer; (**c**) micrograph of the finished APD devices.

The epitaxial structure we designed for the SAGCM In_0.53_Ga_0.47_As/InP planar APD device is shown in Figure 1a. The structure was grown on a four-inch S-doped InP substrate (n~1 × 10^18^ cm^−3^) by MOCVD in an Epihouse device and is comprised of a 0.5-µm-thick Si-doped InP buffer layer (n~5.9 × 10^17^ cm^−3^), a 3.0-µm-thick undoped In_0.53_Ga_0.47_As absorption layer (n~3.5 × 10^14^ cm^−3^), three 0.03-µm-thick Si-doped Ga_x_In_1−x_P_y_As_1−y_ grading layers (n < 1 × 10^15^ cm^−3^), a 0.1-µm-thick Si-doped InP charge layer (n~2.9 × 10^17^ cm^−3^), and, on the top, a 3.5-µm-thick Si-doped cap layer (n~1.8 × 10^14^ cm^−3^). The In_0.53_Ga0._47_As absorption layer conducts photon absorption. The composition x and y of the three Ga_x_In_1−x_P_y_As_1−y_ grading layers [13] were carefully designed to linearly grade the band discontinuity between the In_0.53_Ga_0.47_As absorption layer and the InP charge layer, as indicated by the photo-generated holes. The Si-doping concentration of the InP charge layer was carefully designed to effectively provide an electric field reduction between the InP multiplication layer and the In_0.53_Ga_0.47_As absorption layer to an acceptable level, allowing the electric field in the absorption layer to be low enough to inhibit the band-to-band tunneling current, but high enough to aid in the collection of the photogenerated holes.

After the epi-deposition of the InP wafers, a SiN layer was deposited on the wafer by PECVD. The photolithography technique and RIE etching were used to open the circle or ring area for Zn diffusion. Then, we initiated two Zn diffusions, with different diffusion times, into the InP top layer to form the active region, AGR region, and FGR region, shown schematically in Figure 1b. The central active region experiences twice the Zn diffusion. It has a deeper diffusion depth, while the AGR region and the FGR region experience only the second diffusion, hence, with a shallower diffusion depth. The thickness of the InP multiplication layer is the difference between the thickness of the InP cap layer and the Zn diffusion depth in the central active region. In practice, the diffusion depth was calculated from the punch-through voltage (V_p_) of the designed test devices after the Zn diffusion.

After the two Zn-diffusions, surface of the SiN anti-reflection layer was coated with PECVD. The photolithography technique was used to open the contact rings. Au-Pt-Ti metal contacts and electrodes were deposited and lifted off on the devices. The micrograph of the fabrication is shown in Figure 1c. Reverse dark current I-V measurement was then performed on these APD devices using an HP 4156 parameter analyzer. A 4 mW 1550 nm laser source illuminated the reverse photo current I-V measurement.

## 3. TCAD Simulations

In addition to the device fabrication, we also used Synopsys Sentaurus TCAD software (vL-2016.03) to simulate the diffusion and the electrical properties of the SAGCM APD devices. In the simulation, the radius of the active region was set to 25 μm.

### 3.1. Setup of Epitaxial Layers

The first step in the TCAD simulation is to generate the SAGCM epi-layer. Some of the main parameters are listed in Table 1. To save the simulation time, the substrate thickness was set to 2 µm, which is much larger than the expected depletion region in the simulation.

### 3.2. Diffusion and Electrical Properties Analysis

After these layers were set, the diffusion processes were performed. For the diffusion, we adopted the Fermi model which could be described as:

(1) $$\frac{\partial C_{A}}{\partial t}=\nabla \cdot \sum_{X,c}D_{AX^{c}}{\left(\frac{n}{n_{i}}\right)}^{-c-z}\nabla \left[C_{A}^{+}{\left(\frac{n}{n_{i}}\right)}^{z}\right].$$

where *c* is the charge state of the point defect; *z* is the charge state of dopant A; $C_{A}^{+}$ is the active portion of *C_A_*; *X* is either interstitial or vacancy; and $D_{AX^{c}}$ is the effective diffusivity of the dopant point-defect pairs at charge state *c*. We used the parameters referred to in the work of [14] to generate the diffusion regions, including the central region, AGR region, and FGR region, by two diffusion processes.

After diffusion, a passivation layer and a contact were applied. To simulate the breakdown behavior, we adopted the Okuto–Crowell model [15] to calculate the ionization coefficient in the multiplication region:

(2) $$\alpha \left(F_{ava}\right)=a\cdot \left(1+c\left(T-T_{0}\right)\right)F_{ava}^{\gamma }\exp\left[-{\left(\frac{b\left[1+d\left(T-T_{0}\right)\right]}{F_{ava}}\right)}^{\delta }\right]$$

and the Hurkx field-enhancement model [16], a model that considers trap-assisted tunneling enhanced SRH recombination current, to calculate the dark current before avalanche breakdown.

## 4. Results and Discussions

In this work, we investigate the designs of AGR diffusion depth, the spacing between the AGR ring and FGR ring, and the ring width of FGR in a simple dual diffused SAGCM InGaAs/InP APD device. The epilayer structure is shown in Figure 1a. For the smallest APD device, the radius of the active region is 50 µm, and the width of the AGR ring is 25 µm. In the TCAD simulation, the active region’s radius is shortened to 25 µm to save the run time. The 25 µm radius was selected after a study on the dependence of breakdown behavior on the active region radius. When the radius is equal to or larger than this value, the breakdown voltage becomes radius independent, indicating that the radius is large enough to consider the edge effect. The rest of the structure, i.e., the AGR and FGR, are kept the same as those of the actual devices.

### 4.1. Diffusion Depth of Attached Guard Rings

**Figure 2:**
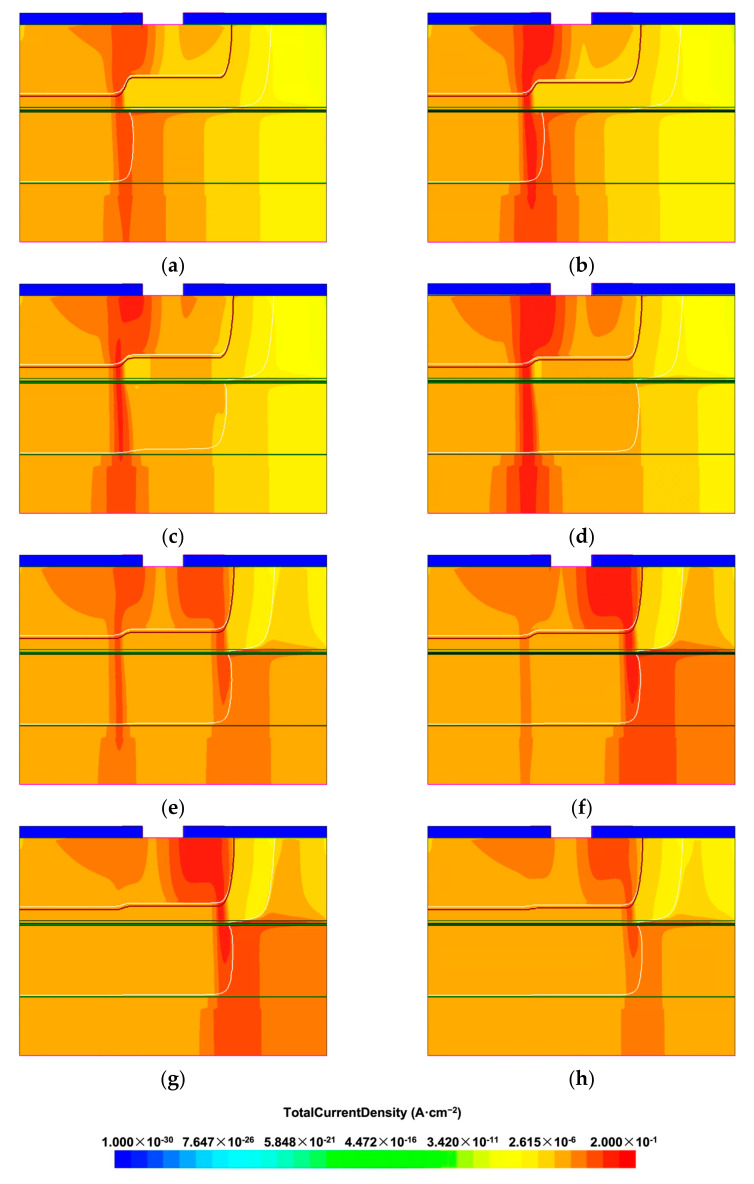
TCAD simulation of current density at breakdown voltage of devices with different AGR depth: (**a**) AGR depth of 2.23 µm; (**b**) AGR depth of 2.44 µm; (**c**) AGR depth of 2.61 µm; (**d**) AGR depth of 2.73 µm; (**e**) AGR depth of 2.77 µm; (**f**) AGR depth of 2.81 µm; (**g**) AGR depth of 2.87 µm; (**h**) AGR depth of 2.95 µm. The diffusion depth of active region is 3.04 µm. The color bar for (**a**–**h**) is displayed below the figures. Two blue rectangles on the top of (**a**–**h**) represent the SiN passivation layer, and the contact is placed between them.

Figure 2 shows the dark current distribution at the breakdown voltage of APD devices with different AGR diffusion depths, simulated by TCAD. The diffusion depth in the active region is 3.04 µm. As shown in Figure 2a–d, for cases of AGR depth from 2.23 µm to 2.73 µm, the current clearly concentrates around the active region’s edge. However, the figures also reveal a current gradually developing in the AGR region with increasing AGR depth. In Figure 2e,f, for the cases of AGR depth = 2.77 µm and 2.81 µm, we can see that the devices show two breakdown current paths located at the edge of the central active region and the edge of the AGR region, respectively. Comparing the current distribution in these two figures, we can find that the current gradually shifts from the edge of the active region to the AGR edge when the AGR depth increases. As can be seen in Figure 2g,h, for the cases with deeper AGR depth, the current path at the edge of the central region ceases, and the breakdown occurs at the AGR edge.

**Figure 3:**
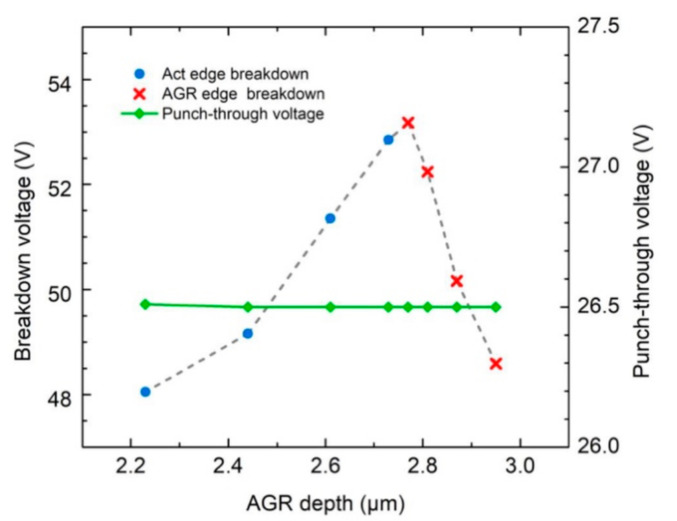
TCAD simulated breakdown voltage and punch-through voltage as functions of AGR depth. The punch-through voltage is independent of AGR depth.

Figure 3 shows the breakdown voltage extracted from the simulations shown in Figure 2a–h. The blue dots represent the cases in Figure 2a–d, where the breakdown still occurs at the edge of the central region. In these cases, the breakdown voltage increases with the increasing AGR depth. On the other hand, the red Xs represent the cases in Figure 2e–h, in which the breakdown path at the AGR edge gradually take over the breakdown current. In these four cases, the breakdown voltage decreases with the increasing AGR depth. From Figure 3, it can be seen that the most significant breakdown voltage is located at the boundary of the two regions, i.e., a device with an AGR depth of 2.77 µm. In this case, the two edges reach the breakdown condition nearly simultaneously, indicating that the electric field resulting from the bias voltage is properly distributed in these two edges to achieve the best breakdown behavior. The difference between the diffusion depth in the central and AGR regions is only 0.27 µm. We attribute this slight difference to the steep diffusion front end of the concentration-dependent Zn diffusion [17]. The steep front end causes the edge breakdown to take place at the bottom corner, as shown in Figure 2, and limits the influence of AGR on the junction profile of the bottom corner until the AGR front is very close to the corner. The punch-through voltage of these devices is also shown in Figure 3. Since the multiplication layer thickness under the central active region is fixed, the punch-through voltage is independent of the AGR depth.

**Figure 4:**
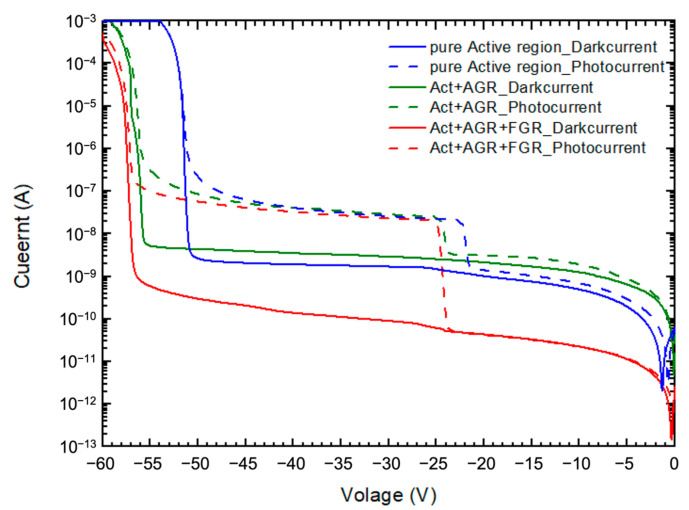
Dark current (solid) and photo current (dash) of APD devices with three different designs: without guard ring (blue, Device A), with AGR only (green, Device B), and with AGR and FGR (red, Device C).

The optimal structure, obtained from Figure 2 and Figure 3, was used to design the diffusion times for the two Zn diffusions performed on our APD devices. Dual diffusions form the active region, with an accumulated depth of 3.04 µm. While the AGR and FGR are created only by the second diffusion, and the depth is 2.77 µm. The dual diffusion also generates a 0.46-µm-thick multiplication layer in the active region. In practice, we arranged test devices to monitor the diffusion depths by measuring their punch-through voltage. However, the punch-through voltage of the finished APD devices in this batch varied from −21.5 V to −25 V. The corresponding variation of dual-diffusion depth is from 3.09 µm to 2.99 µm. Figure 4 shows the typical dark and photo I-V characteristics of our three APD devices fabricated in the same batch, but with different guard ring designs. Red curves represent the I-V behavior of the device with AGR and FGR structure. The diameter of the active region is 100 µm, and the opening widths for AGR and FGR are 25 µm and 4 µm, respectively. The spacing between AGR and FGR is 6 µm. Green and blue curves represent the I-V behaviors of the device with AGR, but without FGR, and the device without any guard rings, respectively. The AGR opening width and active region radius are the same as those of the device with AGR and FGR. As can be seen, all the devices have their dark current at 0.9V_b_ in the level of 10^−9^~10^−10^ A. In addition, the two devices with AGR have larger breakdown voltages than the one without AGR, indicating the advantage of using an AGR structure. The breakdown voltage of the device with AGR and FGR is slightly higher than that of the device with AGR only, suggesting that the effect of FGR is minor. In this work, the breakdown voltage, V_b_, is defined as the bias voltage when the dark current reaches 100 µA.

**Figure 5:**
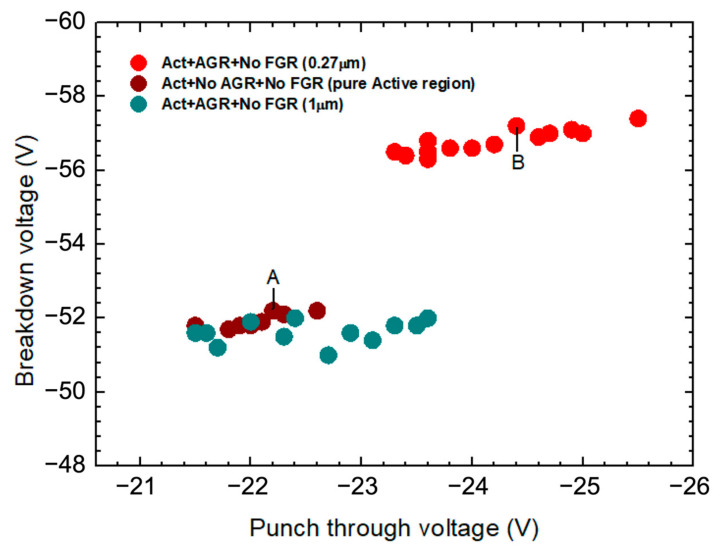
Breakdown voltages of devices with optimal AGR (red), without AGR (brown), and with shallower AGR (green), as functions of punch-through voltages. The depth of the optimal AGR is shallower than that of the central active region by 0.27 µm, while the depth of the shallower AGR is shallower than that of central active region by 1 µm. The brown circle marked “A,” and the red circle marked “B” are device A and B, respectively, in Figure 4.

Figure 5 shows the breakdown voltage as a function of punch-through voltage for three groups of APD devices. Red and brown circles represent the groups of devices with and without AGR, respectively. These two groups of devices were fabricated in the same batch with the aforementioned optimal diffusion conditions. As can be seen, the breakdown voltage slightly increases with the increasing punch-through voltage, which is the expected behavior of APD devices. In our structure, devices with thinner multiplication layers have lower punch-through voltage and breakdown voltage. The slight variation in punch-through voltage is relevant to the uniformity of the epi-growth and Zn diffusion. Comparing the trends of red and brown circles, the breakdown voltages of the devices with AGR are ~5 V higher than those without AGR. Another group of devices, represented by dark green circles, was fabricated in another batch, with different diffusion parameters. Their AGR depth is 2 µm. The breakdown voltages of this group of devices, as shown in Figure 5, are very close to those of devices without AGR in the batch with optimal diffusion conditions. Since the 2 µm AGR depth is far above the bottom corner of the active region, this finding confirms our argument that AGR does not affect the breakdown behavior until its depth is very close to the bottom edge of the active region.

### 4.2. Design of Floating Guard Rings

Based on the optimal AGR structure, we turn to the FGR structure. In the design of FGR, two parameters, the spacing between the opening widows of AGR and FGR in photomasks for Zn diffusion, hereafter called AF spacing, and the width of the FGR’s opening window, are tuned to study how these two design parameters affect the breakdown voltage. In this study, we designed different FGR widths, varying from 2 to 6 µm, and six different AF spacings, ranging from 3 µm to 8 µm. In addition, the devices without FGR are also included in the comparative study.

**Figure 6:**
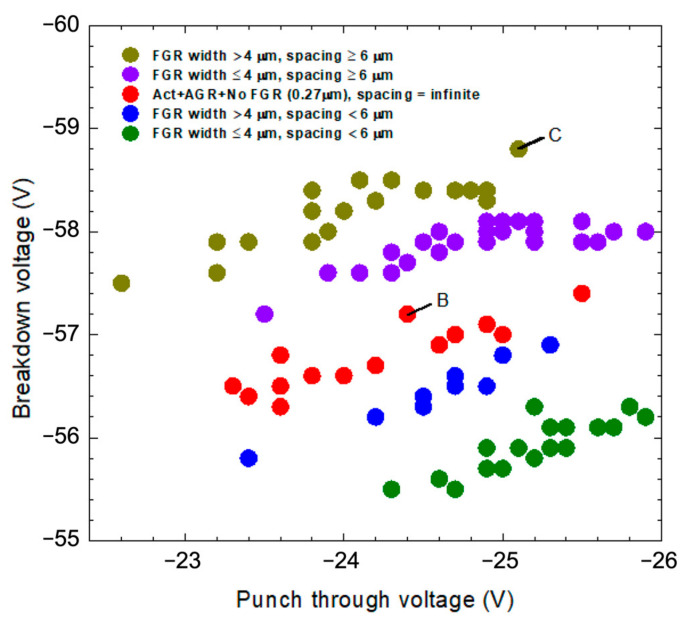
Breakdown voltage and punch-through voltage distribution for devices with different FGR structures. The red circle marked “B,” and the olive-green circle marked “C,” represent devices B and C, respectively, in Figure 4.

Figure 6 shows the breakdown voltage of the APD device with different FGR designs as a function of punch-through voltage. The results are divided into five groups, according to their breakdown behaviors. The olive-green dot represents the group with an FGR width > 4 µm and an AF spacing ≥ 6 µm, with the highest breakdown voltage. The group of devices with an FGR width ≤ 4 µm and an AF spacing ≥ 6 µm, represented by purple dots, has the second-highest breakdown voltage. Thus, we can see the group of devices without FGR, represented by red dots, lies below those represented by purple dots. The other two groups of devices with an AF spacing < 6µm have smaller breakdown voltage than those without AGR. In these two groups, the example with an FGR width > 4 µm has a higher breakdown voltage than the other.

The findings from Figure 6 suggest that AF spacing has a critical value of 6 µm. When the spacing is above the critical values, the devices show better breakdown behaviors than the devices without FGR. On the other hand, when the spacing is below the critical value, the breakdown voltages are worse than those of the devices without FGR. Whether the AF spacing is above or below the critical value, a larger FGR width results in higher breakdown voltages. However, the effect of the FGR width seems less significant than that of AF spacing.

**Figure 7:**
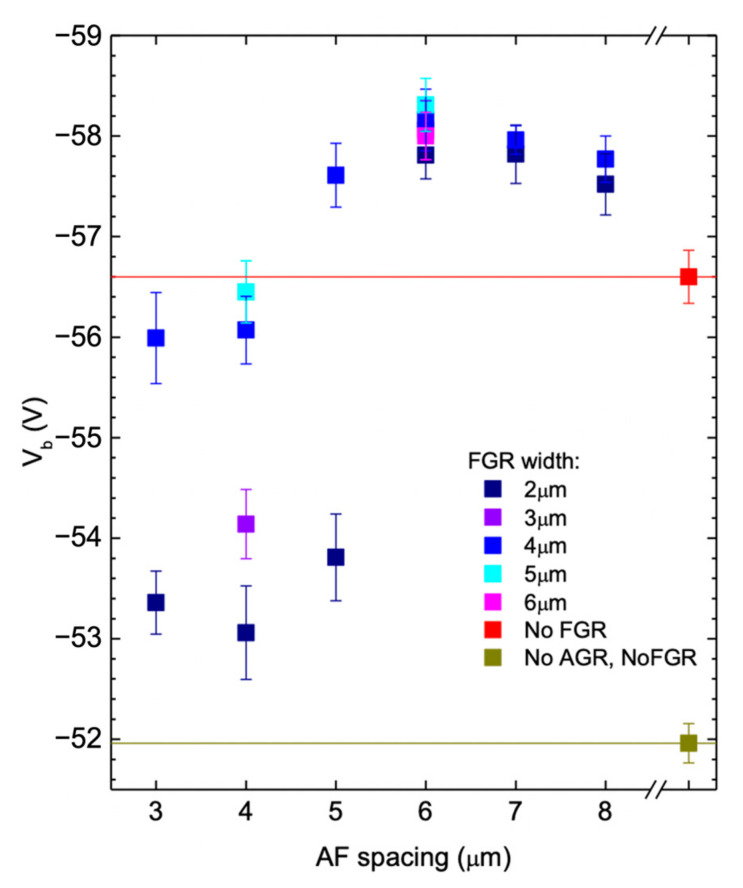
Breakdown voltage as a function of AF spacing for devices with different FGR widths. Red square and red line represent the devices with AGR, but without FGR. Brown square and brown line represent the devices without the guard ring structure.

To further understand the relationship between the breakdown voltage and the FGR design, Figure 7 shows the breakdown voltage as a function of AF spacing. For each AF spacing, results with different FGR widths are shown with error bars. The red horizontal line, representing the average breakdown values of devices without FGR, separates the result into two categories. For AF spacing less than 4 µm, all devices have breakdown voltages below the red line, the breakdown voltage of devices without FGR. When the AF spacing is 5 µm, the devices are divided into two groups, one above the red line and the other below the red line. The group shows better breakdown voltage with a wider FGR width of 4 µm, suggesting that a wider FGR width provides better breakdown voltage. A similar trend can also be observed in other groups. When AF spacing goes over 6 µm, regardless of FGR width, all devices have exhibit better breakdown voltages than the devices without FGR. However, the average breakdown voltage still increases slightly with the widening of the FGR width. From this figure, one can determine a critical value for AF spacing, which is 6 µm. From the figure, we also found that the effect of AF spacing on the breakdown behavior seems to be more prominent than that of FGR width.

Notice that the aforementioned FGR width and AF spacing are the nominal distances on the photomasks. However, thermal diffusion is a random walk process of Zn atoms, and lateral diffusion beneath the SiN mask is inevitable. The lateral diffusion shortens the distance between AGR and FGR and, and, even worse, it may short-circuit the AGR and FGR and fail to function in the FGR.

**Figure 8:**
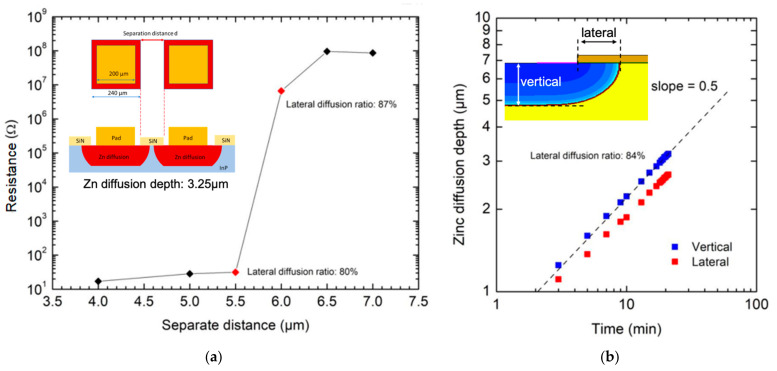
Lateral diffusion: (**a**) resistance as a function of separate distance between square pads. The inset shows the schematic diagram of test pads designed for resistance measurement. (**b**) Vertical diffusion depth and lateral diffusion length as functions of diffusion time. The results come from TCAD simulation using the Fermi model. The definitions of vertical diffusion depth and lateral diffusion length are shown in the inset.

To evaluate the lateral diffusion length in our diffusion condition, we designed a test kit consisting of pairs of square openings with different spacings for Zn diffusion. After the diffusion, metal contacts were deposited on the openings for I-V characterization. Figure 8a shows the resistance as a function of spacing. A schematic diagram of a test pair is shown in the inset of this figure. As can be seen, the resistance possesses a distinct transition from ~20 Ω to 10^7^~10^8^ Ω, when the separation distance is between 5.5 and 6 µm. The growth suggests a lateral length of 2.7~3 µm. The vertical diffusion depth is determined from the devices’ punch-through voltage, and it is 3.25 µm. The experiment indicates that the lateral length is ~0.87 of the diffusion depth.

Figure 8b shows the results from TCAD simulation of vertical diffusion depths and lateral diffusion length as functions of diffusion time. The Fermi model was used for the simulations. Both vertical diffusion depth and lateral diffusion length are proportional to the square root of the diffusion time. Their results, represented by blue and red squares, are in parallel in the log–log plot, suggesting that the lateral diffusion length is ~0.84 of the vertical diffusion depth, which is close to the ratio obtained from the experimental test.

Since the diffusion depth of the AGR is ~2.8 µm, the estimated lateral diffusion length is ~2.5 μm. Therefore, the AF spacing will be reduced by ~5 µm by the lateral diffusions from the inner and outer sides. For the AF spacing of 2 µm to 4 µm, the FGR may connect to the AGR directly, losing its utility. When the AF spacing is 6 µm or larger, the FGR and AGR are effectively separated to allow the depletion region of FGR to affect the edge of AGR, without the physical contact of AGR and FGR. As shown in Figure 6, the two groups of devices with AF spacing ≥ 6 µm have a higher breakdown voltage than those without FGR, and the same result can also be seen in Figure 7.

**Figure 9:**
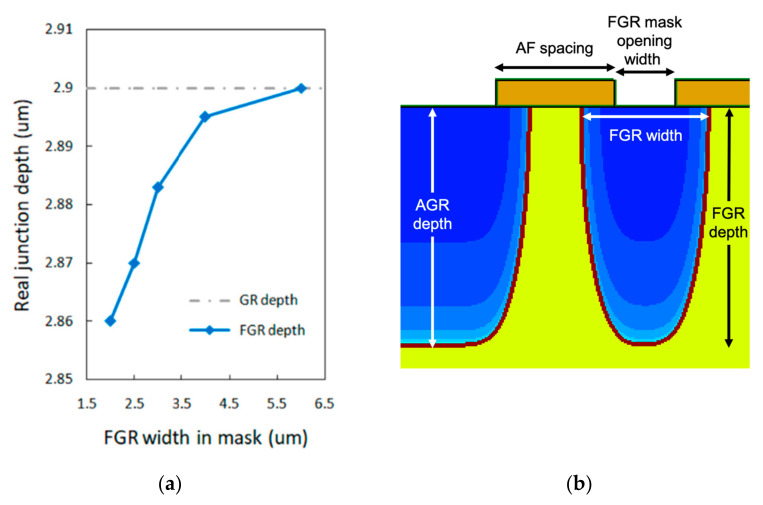
(**a**) Simulation result of diffusion depth for different mask width of FGR. In the simulation, the diffusion time is fixed. (**b**) Parameter definitions of FGR and AGR in this work are shown in the device cross-section.

We also found that when the size of the diffusion opening is close to the lateral diffusion length or vertical diffusion depth, the limit of the Zn source may affect the diffusion depth. We performed TCAD simulations to study this issue. Figure 9a shows the calculated vertical diffusion depth as a function of the opening width of FGR. All data points were created using the same diffusion time. Figure 9b shows a cross-section of the AGR and FGR structures, in which the lengths and positions of actual FGR width, FGR mask width, and FGR depth after diffusion are indicated. In this simulation, the much wider diffusion opening results in an AGR depth of 2.9 µm. In contrast, the vertical diffusion depth of the FGR decreases when the FGR width decreases from 6 µm to 2 µm. This opening size effect can explain the finding obtained in Figure 7, in which the breakdown voltage increases with the increasing FGR width. In Section 4.1, we have shown that the breakdown occurs around the bottom corner of the AGR region. For the FGR with a wider opening width, the diffusion depth is closer to that corner, allowing the FGR to reduce the electric field around the corner and thus improve the breakdown behavior, even though the improvement is minor.

**Figure 10:**
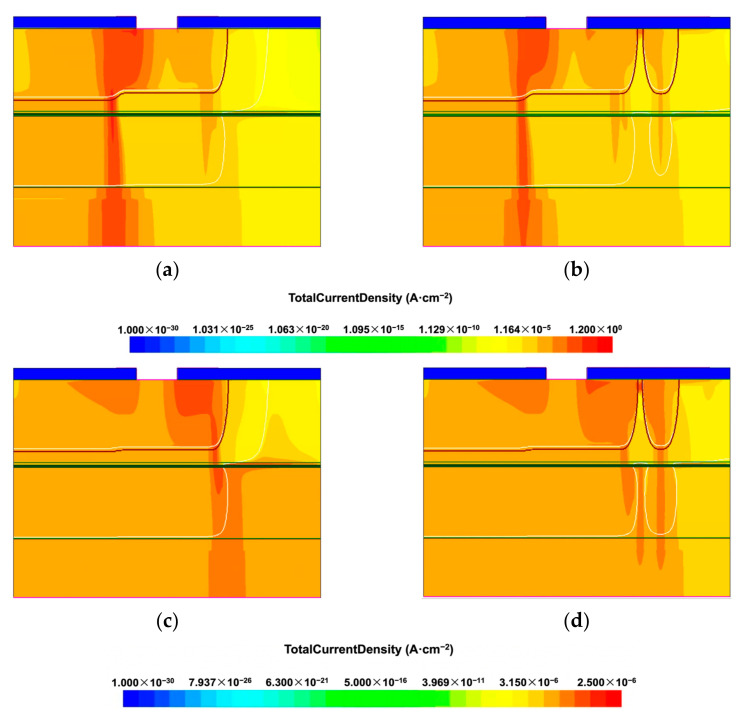
Current density distribution in the device with different guard ring structures: (**a**) AGR with a diffusion depth of 2.73 μm only; (**b**) AGR and FGR with the same diffusion depth, 2.73 μm, with the AF spacing of 6 μm; the color bar for (**a**,**b**) is displayed below the figures. (**c**) AGR with a diffusion depth of 2.95 μm only; (**d**) AGR and FGR with the same diffusion depth, 2.95 μm, with the AF spacing of 6 μm; the color bar for (**c**,**d**) is displayed below the figures. In (**a**,**b**), the bias voltage is −52.85 V. The breakdown voltages for the device in (**a**,**b**) are −52.85 V and −52.96 V, respectively. In (**c**,**d**), the bias voltage is −48.59 V. The breakdown voltages for the device in (**c**,**d**) are −48.59 V and −48.68 V, respectively.

TCAD simulation was also performed to understand the effect of FGR on the current density distribution at the breakdown voltage of the devices. Figure 10a,b shows the current density distribution in the devices, without and with FGR, respectively. The diffusion depth of their AGR is 2.73 µm, and the AF spacing is 6 µm. For the device without FGR, the breakdown current concentrates at the edge of the active region, as seen in Figure 10a. When the FGR is used, the current at the active region’s edge decreases, and the currents in AGR and FGR increase, as shown in Figure 10b. The use of FGR increases the breakdown voltage from −52.85 V to −52.96 V. Figure 10c,d also shows the current density distribution in devices, without and with FGR, respectively. The diffusion depth of the AGR is 2.95 µm, and the AF spacing is 6 µm. In Figure 10c, the breakdown current concentrates at the edge of the AGR. The use of FGR significantly decreases the current at the AGR edge and increases the breakdown voltage from −48.59 V to −48.68 V.

The simulation indeed shows that the use of FGR improves the breakdown behavior. However, the amount of improvement in the breakdown voltage is much lower than that of the experimental results we observed in Figure 7. We attribute the discrepancy largely to the fabrication process of the experimental devices. Basically, the TCAD model can fit the vertical diffusion depth quite well. However, the protection of FGR is obtained through the lateral depletion region. Because the lateral diffusion is strongly dependent on the surface vacancy density, which could be drastically affected by the preparation of the SiN diffusion mask, and other processing steps, we believe that the experimental devices could have a softer lateral diffusion front, and thus show better improvement.

### 4.3. Gain and Quantum Efficicncy

In order to estimate the gain for the experimental devices [18], their dark currents (I_D_) and photocurrents (I_P_) were measured. The current differences were obtained by subtracting I_D_ from I_P_. Afterward, we averaged these current differences from −30 V to −45 V to obtain I_diff−uni_, which indicated the flat part of I-V curves in measurement. The gain was calculated by:

gain = (I_P_ − I_D_)/I_diff−uni_ (3)

**Figure 11:**
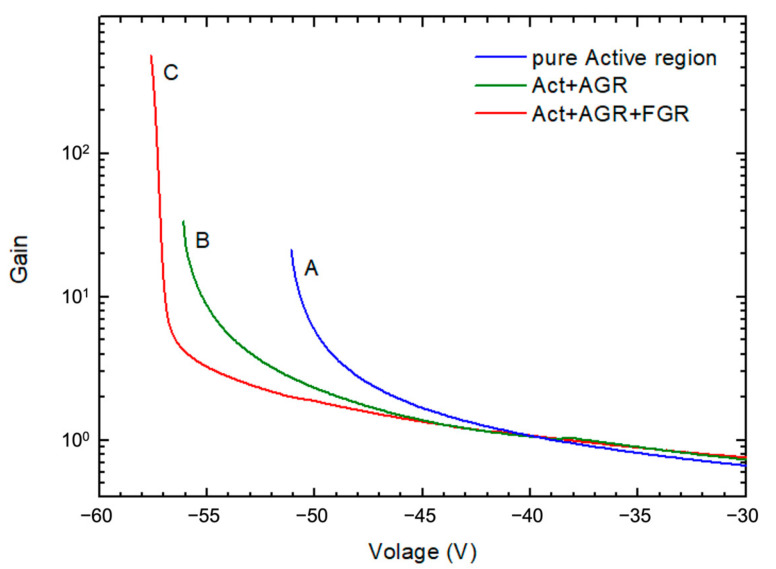
The gain curves obtained from the I-V curves shown in Figure 4. A, B, and C represent device A, B, and C in Figure 4.

The gain calculated from Figure 4 is shown in Figure 11. The gain increases with the increasing bias voltage. The green curve has a higher gain at 0.98 V_b_ than the blue curve, showing the advantage of applying AGR to a pure active region; the red curve has the highest gain at 0.98 V_b_, proving that FGR can not only improve breakdown voltage, but can also further enhance gain.

**Figure 12:**
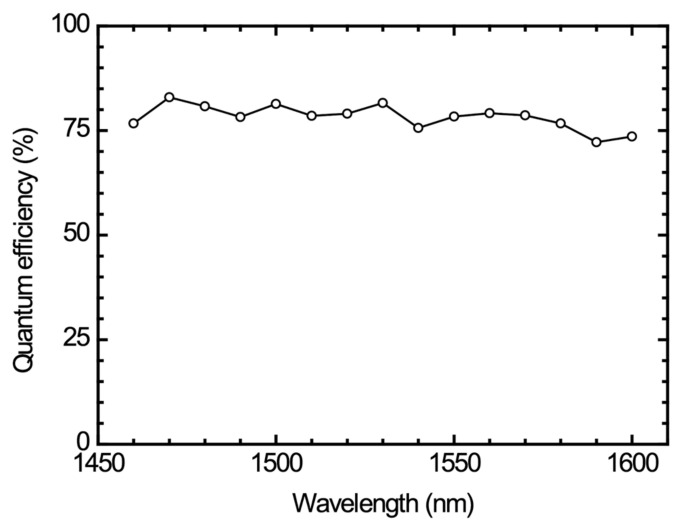
Quantum efficiency as a function of wavelength.

We used the HP 8168C tunable laser source and the HP 4145B parameter analyzer to measure the quantum efficiency of our APD devices. The result is shown in Figure 12. In the tunable wavelength range of 1460 nm to 1600 nm, the quantum efficiency is around 75%.

### 4.4. Device Performance Comparison

In Table 2, we compare the device performances with results reported in the literature for InP APD devices with various periphery protection designs. Our devices demonstrate lower dark current and better gain near the breakdown voltage. The breakdown voltage in the literature is usually the result of the final devices with their protection structures, and most of them did not provide the data for devices without protection structures. Since the breakdown voltage is mainly determined by the thickness of the multiplication layer and cannot directly represent the efficacy of the protection structure, we only list the values for reference.

## 5. Conclusions

In this work, we have demonstrated how to improve the performance of simple planer 1550-nm SAGCM In_0.53_Ga_0.47_As/InP APD using double Zn diffusions by providing it with properly designed AGR and FGR. The parameters we studied include the diffusion depth of AGR, the spacing between AGR and FGR, and the opening width of FGR. Through TCAD simulation, we found an optimal AGR diffusion depth of 2.77 µm for a 3.04 µm deep active region. The AGR depth is shorter than the depth of the central active region by 0.27 µm. When AGR diffusion depth is shorter than this optimal depth, the breakdown takes place at the edge of the central active region, and the AGR is ineffective. However, when the AGR diffusion depth is deeper than the optimal depth, the breakdown shifts to the edge of the AGR, and the breakdown voltage decreases with the increasing diffusion depth.

For the design of AF spacing, we found a critical value of 6 µm. When the AF spacing is larger than 6 µm, the breakdown voltage is higher than that of the device without FGR, regardless of the FGR opening width. The critical value is relevant to the lateral diffusion. In our Zn diffusion, the estimated lateral diffusion length is 2.41 µm. As a result, when the AF spacing is shorter than the critical value, which is about twice the lateral diffusion length, the FGR is short-circuited to the AGR and loses its function, causing poor breakdown behavior. FGR width also affects breakdown behavior; a wider FGR opening width results in a higher V_b._ However, the effect of FGR width is less significant than that of AF spacing. TCAD simulation was also performed to show the effect of FGR, and it exhibited less pronounce improvements, which could be due the discrepancy between the calculated and experimental diffusion profiles.

## Figures and Tables

**Table 1:** Epi-layer information for the simulation.

Layer Name	Material	Dopant Level (cm^−3^)	Thickness (µm)
Cap layer	Si-doped InP	1.76 × 10^14^	3.5
Charge layer	Si-doped InP	2.9 × 10^17^	0.1
Grading layer	InGaAs		0.1
Absorption layer	InGaAs		3
Buffer layer	Si-doped InP	5.85 × 10^17^	0.5
Substrate	S-doped InP	2 × 10^18^	2

**Table 2:** Comparison of our photodiode with those in the literature.

Reference	**This work**	[6]	[7]	[8]	[10]
Epi	**SAGCM**	-	SAM	SAM	SAGCM
Multiplication layer	**InP**	InP	InP	InP	InP
Diffusion	**Zn**	Be implantation	Zn	Zn	Zn
Periphery design	**AGR+FGR**	PLEG (deep AGR + shallow AGR)	Double Zn diffusion + FGR (AGR + FGR)	Primary diffusion well + FGR (AGR + FGR)	FGR
Dark current	**10^−9^~10^−10^ A** **@0.9 V_b_**	2·10^−8^ A @0.9 V_b_	1.1·10^−8^ A @0.9 V_b_	10^−8^ A level @0.9 V_b_	10^−8^ A level @0.95 V_b_
V_b_	**55.4~58.8 V**	102.5~117.5 V	70~85 V	Around 38 V	32.5~36 V
Diameter of light receiving area	**100 µm**	50 µm	75 µm	-	50 µm
Overall gain	**With best device** **up to 400 @0.98V_b_**	-	25@V_b_	>10	10@V_b_

## Data Availability

Data sharing not applicable.
